# Machine Learning Approach to Comparing Fatty Acid Profiles of Common Food Products Sold on Romanian Market

**DOI:** 10.3390/foods12234237

**Published:** 2023-11-23

**Authors:** Florina-Dorina Covaciu, Camelia Berghian-Grosan, Ariana Raluca Hategan, Dana Alina Magdas, Adriana Dehelean, Gabriela Cristea

**Affiliations:** National Institute for Research and Development of Isotopic and Molecular Technologies, 67-103 Donat Street, 400293 Cluj-Napoca, Romania; florina.covaciu@itim-cj.ro (F.-D.C.); camelia.grosan@itim-cj.ro (C.B.-G.); ariana.hategan@itim-cj.ro (A.R.H.); alina.magdas@itim-cj.ro (D.A.M.); adriana.dehelean@itim-cj.ro (A.D.)

**Keywords:** fatty acids, food products, boxplot diagram, GC-FID, machine learning

## Abstract

Food composition issues represent an increasing concern nowadays, in the context of diverse food commodity varieties. The contents and types of fatty acids are a constant preoccupation among consumers because of their reflections of nutrition and health problems. This study aims to find the best tool for the rapid and reliable identification of similarities and differences among several food items from a fatty acid profile perspective. An acknowledged GC-FID method was considered, while, for a better interpretation of the analytical results, machine learning algorithms were used. It was possible to develop a recognition model able to simultaneously differentiate, with an accuracy of 79.3%, nine product types using the bagged tree ensemble model. The low number of samples or some similarities among the classes could be responsible for the wrong assignments that occurred, especially in the biscuit, wafer and instant soup classes. Better accuracies values of 95, 86.1, and 97.8% were obtained when the products were grouped into three categories: (1) sunflower oil, mayonnaise, margarine, and cream cheese; (2) biscuits, cookies, margarine, and wafers; and (3) sunflower oil, chips, and instant soup.

## 1. Introduction

Fatty acids play a crucial role in nutrition and overall health. They are the fundamental components of triglycerides and the primary constituents of fats and oils, comprising three main classes: saturated, monounsaturated, and polyunsaturated fatty acids. The fatty acid composition of foods can vary depending on factors such as the plant or animal source, processing methods, and cooking techniques [1]. The specific amounts of different fatty acids in a particular food can also range depending on the variety of the food product, brands, recipes, and manufacturing processes [2].

While dietary lipids offer benefits such as enhancing the absorption of fat-soluble vitamins, providing concentrated energy, insulation for heat conservation, acting as a lubricant for bodily functions, and imparting flavor to food, the excessive consumption of specific fatty acid classes has been associated with an elevated risk of obesity, liver disease, and increased serum cholesterol levels, potentially leading to cardiovascular diseases [3,4]. The majority of this excessive intake is believed to originate from commercially processed foods, including snacks and restaurant meals, each containing fluctuate fatty acid compositions [5,6]. In contrast to saturated fats, unsaturated oils like olive oil are considered healthier and less likely to cause adverse health effects. Among fatty acid classes, saturated fats (SFAs) contribute more significantly to obesity compared to monounsaturated (MUFAs) and polyunsaturated fats (PUFAs) [7]. Furthermore, MUFAs and PUFAs are absorbed more readily than SFAs, which has a slower absorption rate. The recommended daily intake of SFAs is less than 10% of total calories, starting at the age of two [8]. Nutrition interventions aim to alter dietary fatty acids to minimize the risk of cardiovascular diseases [9].

In recent years, there has been a notable increase in studies combining classic analytical techniques with various machine learning (ML) algorithms, a strategy that enhances the reliability of results, both qualitatively and quantitatively. The applications of ML in food science have expanded significantly [10,11]. For instance, ML algorithms have been employed in food authentication by analyzing specific markers in nutritional studies to predict associations between obesity and demographics, like age, body mass index, and gender [12]. They have also been used to differentiate products based on species, geographical origin, and production method [13], as well as to forecast the quality of plant-based foods [14]. In recent years, significant attention has been also paid to food adulteration detection by reliable methods that combine common analytical techniques and various traditional or deep learning techniques [15]. Some studies have also explored the possibility of using the fatty acid composition for adulteration detection in various oils; thus, the methodology obtained by combining the gas chromatographic technique with ML-based algorithms, such as support vector machine (SVM), k-nearest neighbor (k-NN), or decision tree (DT), and a neural network algorithm for regression has proved to be effective for the identification of fraudulent olive oils mixed with cottonseed, canola, or soybean oils [16]. A comprehensive study involving ten types of edible oils, a huge number of samples, and a large dataset containing 18 different fatty acids obtained by gas chromatography with flame ionization detector (GC-FID) highlighted the utility of using a deep learning model for the adulteration detection of edible oils [17].

In fatty acid analysis, GC-FID involves the separation of individual fatty acids from a complex mixture and their subsequent detection and quantification using a flame ionization detector. The resulting chromatogram provides information about the types and relative abundances of fatty acids present in the sample [18]. By leveraging ML algorithms and boxplot diagrams to analyze fatty acid profiles determined by GC-FID, it becomes possible to identify subtle similarities and differences in order to accurately discriminate among multiple food products. This aids in the quality control, authenticity verification, product development, and overall understanding of the fatty acid composition of different food items [19,20].

This study tests the use of ML algorithms for the rapid and reliable discrimination of food products considering their fatty acid profiles. Thus, the main objectives of the present study are: (i) to determine the fatty acid profile in different food products using the GC-FID technique, and (ii) to explore the possibility given by ML for the identification of distinct subtle changes that occur in the fatty acid profiles of different food products. The application of ML algorithms to GC-FID data holds promise for improving the accuracy, efficiency, and depth of analysis in assessing the fatty acid profiles of food products.

## 2. Materials and Methods

### 2.1. Samples Description

A total of 150 food samples of different types and brands sold in Romanian supermarkets were tested. The samples were commercialized by various manufacturers existing in the market, and the sample distribution was as follows: sunflower oil (*n* = 20); mayonnaise (*n* = 20); margarine (*n* = 20); cream cheese (*n* = 20); instant soup (*n* = 20); chips (*n* = 20); cookies (*n* = 20); biscuits (*n* = 5); and wafers (*n* = 5).

### 2.2. Fatty Acid Analysis

The sample preparation protocol is described in detail in our previous work [21]. The entire set of 150 samples was experimentally processed through a stage of hydrolysis of the fat, followed by a stage of methylation of the obtained fatty acids. The resulting samples, after the preparation step, were used to determine the fatty acids by GC-FID.

GC-FID analysis was carried out by a Trace GC Ultra gas chromatograph equipped with a flame ionization detector from Thermo Electron Corporation (Milan, Italy). FAME separation was achieved on a DB-FATWAX ultra inlet capillary column (30 m × 0.32 mm i.d., 0.25 µm, Agilent Technologies (Santa Clara, CA, USA)). The injector and detector temperatures were maintained at 250 °C. The oven parameters were set as follows: an initial temperature of 50 °C (held for 2 min) was increased at 4 °C/min to 220 °C and held for 15 min. Helium was used as the carrier gas at a flow rate of 1.8 mL/min, and 1 µL of the sample was injected using a split ratio of 1:100. Identification of the compounds was performed by comparison of their retention times with those of a standard mixture, namely 37 Component FAME Mix from Supelco (Bellefonte, PA, USA). In addition, the *trans* fatty acids were identified with an individual standard also purchased from Supelco. The results were expressed as the relative percentage of each fatty acid, calculated based on the chromatographic peak area.

### 2.3. Data Processing

The distribution of the experimental data, encompassing the GC-FID analyses (concentrations of *cis*/*trans* isomers of fatty acids) of a total set of 150 samples of food products was evaluated through box plots. ML investigations were carried out on the GC-FID results, using the Classification Learner app from MATLAB R2018b (MathWorks Inc., Natick, MA, USA) and various algorithms, such as trees, linear or quadratic discriminants, support vector machines (SVM), k-nearest neighbors classification (kNN), or ensemble.

## 3. Results

Nowadays, food composition and safety are a continuing concern of all involved groups, from authorities and control laboratories to the final beneficiary represented by consumers. Moreover, there are some food commodities that are considered to represent a threat to an equilibrated diet and healthy life in the general perception. This perception is mainly fed by public opinions and not always expressed from the scientific research side. For this reason, this study compares three categories of samples that have in common a relevant fatty acid fingerprint. The first product category comprises four food varieties: sunflower oil, mayonnaise, margarine, and cream cheese; the second one contains biscuits, cookies, margarine, and wafers; the third one is formed by sunflower oil, chips, and instant soup. In the second and third groups, the main sources of fatty acids, namely margarine and sunflower oil, respectively, were included in order to detect their presence in the subsequent products.

The fatty acid (FA) profiles of these nine industrially produced foods, purchased from the Romanian market, were investigated using the gas chromatographic technique, GC-FID. This method was used in our previous study that investigated the variations in the compositions of fatty acids in breast milk during the first 6 months of lactation [21].

The fatty acid profiles of the nine investigated fat-based food classes (biscuits, chips, cookies, cream cheese, mayonnaise, margarine, wafers, instant soup, and sunflower oil) revealed great variation in the identified fatty acids concentrations, both inside each category and among the classes. Considering this aspect but also the large number of data that have been recorded for the 150 samples, the necessity of using a boxplot diagram for a clearer visualization of the results appears to be evident. This chart is a graph that encapsulates the most important statistical characteristics of some frequency distributions to provide a better understanding and comparison, graphically reflecting the summary by the five values of a distribution: the minimum value, the first quartile (or lower quartile), the median, the third quartile (or upper quartile), and the maximum value. The analysis of the boxplot graph also indicates the presence of outliers, those marked with a circle, which represent the extreme values of the dataset [22].

**Figure 1:**
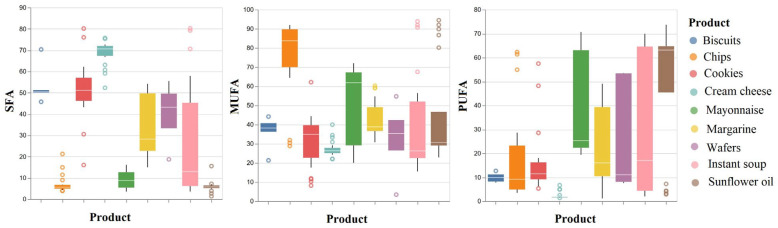
Distribution of the fatty acid concentration values obtained by GC-FID in the investigated food products.

In this context, a boxplot diagram (Figure 1) was used to visualize the distribution of fatty acids grouped in saturated fatty acids (SFAs), monounsaturated fatty acids (MUFAs), and polyunsaturated fatty acids (PUFAs) in relation to the nine types of investigated food products.

The SFA distribution is: asymmetric to the right (low scores predominate) in the case of chips (3.87–21.18%, average of 7.08%), margarine (14.92–54.12%, average of 34.66%), and instant soup (3.58–80.23%, average of 27.70%); asymmetric to the left (high scores predominate) in the samples of cream cheese (52.31–75.70%, average of 68.73%) and wafers (18.68–55.44%, average of 40.03%); and symmetrical in the biscuit samples (45.74–70.32%, average of 53.60%), cookies (16.01–80.16%, average of 51.61%), mayonnaise (3.53–16.09%, average of 9.04%), and sunflower oil (1.32–15.52%, average of 5.93%).

The MUFA distribution is: asymmetric to the right (low scores predominate) in the case of margarine samples (30.75–60.28%, average of 42.93%), instant soup (15.53–93.80%, average of 41.31%), and sunflower oil (22.91–94.49%, average of 44.68%); asymmetric to the left (high scores predominate) in the chip samples (28.85–91.89%, average of 74.84%), cookies (8.15–62.12%, average of 31.34%), and mayonnaise (20.01–72.00%, average of 50.26%); and symmetrical in the samples of biscuits (21.28–44.15%, average of 36.12%), cream cheese (21.99–39.93%, average of 27.39%), and wafers (3.39–54.72%, average of 32.44%).

The PUFA distribution is: asymmetric to the right (low scores predominate) in the samples of chips (3.47–62.24%, average of 17.37%), cookies (5.28–57.46%, average of 16.15%), mayonnaise (19.42–70.62%, average of 39.75%), margarine (1.12–48.98%, average of 21.81%), wafers (7.56–53.51%, average of 26.70%), and instant soup (1.99–69.91%, average of 30.42%); asymmetric to the left (high scores predominate) in the sunflower oil samples (2.85–73.68%, average of 49.24%); and symmetrical in the biscuit samples (7.78–12.63%, average of 9.91%) and cream cheese (1.36–6.66%, average of 2.29%).

**Figure 2:**
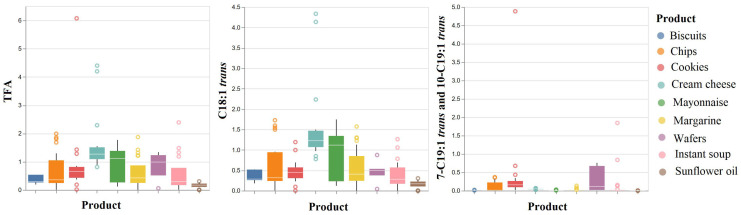
Distribution of *trans* fatty acid (TFA) concentration values in the investigated food products obtained by GC-FID.

From the analysis of the graph presented in Figure 2, the distribution of TFAs is: asymmetric to the right (low scores predominate) in the case of the biscuit sample (the concentration varied between 0.19–0.54%, with an average of 0.37%), chips (0.01–1.99%, average of 0.71%), margarine (0.00–1.87%, average of 0.61%), and instant soup (0.01–2.39%, average of 0.57%); asymmetric to the left (high scores predominate) in the mayonnaise samples (0.12–1.77%, average of 0.94%) and wafers (0.06–1.34%, average of 0.83%); and symmetrical in the samples of cookies (0.02–6.08%, average of 0.91%), cream cheese (0.81–4.40%, average of 1.60%), and sunflower oil (0.00–0.31%, average of 0.15%).

The distribution of vaccenic acid (*t*11-C18:1) is asymmetric to the right (low scores predominate) in the case of the chip samples (the concentration varied between 0.01 and 1.73%, with an average of 0.60%), cream cheese (0.77–4.33%, average of 1.55%), margarine (0–1.57%, average of 0.57%), and instant soup (0–1.26%, average of 0.41%); asymmetrical to the left (high scores predominate) in the biscuit samples (0.18–0.52%, average of 0.35%), mayonnaise (0.12–1.75%, average of 0.92%), and wafers (0.04–0.87%, average of 0.46%); it is symmetrical in the cookie samples (0–1.19%, average of 0.48%) and sunflower oil (0–0.30%, average of 0.15%).

The distribution of *trans*-7-nonadecenoic acid (*t*7-C19:1) and *trans*-10-nonadecenoic acid (*t*10-C19:1) is asymmetric to the right (low scores predominate) in the case of the chip samples (0–0.37%, average of 0.11%), cookies (0.02–4.88%, average of 0.43%), margarine (0–0.13%, average of 0.02%), wafers (0.02–0.76%, average of 0.32%), and instant soup (0–1.85%, average of 0.15%); and symmetrical in the samples of biscuits (0.01–0.02%, average of 0.01%), cream cheese (0.02–0.07%, average of 0.05%), and mayonnaise (0–0.03%, average of 0.01%). The sunflower oil samples appear symmetrical because no *trans*-7-nonadecenoic acid and *trans*-10-nonadecenoic acid were detected.

Even if the exact fatty acid compositions of these nine food products existing on the market can be determined using a chromatographic technique such as GC-FID, and even if this method permits *trans* fatty acid identification, both qualitatively and quantitatively, the obtained databases are very large and require specialized analysis to interpret the results. Moreover, the existence of multiple types of fatty acids and their varying concentrations makes it difficult to assign the samples to a specific class.

In this context, the use of artificial intelligence, through ML algorithms, which proved to be an effective tool for identifying distinct subtle modifications that occur from some food/beverage items to others [23,24,25,26], seems to be necessary. In addition to the huge potential for the detection of food adulteration issues when small quantities of prohibited substances are added to a certain food product, an important advantage offered by this approach consists of the detection of some common compounds that can be found in distinct food commodities. For this reason, ML algorithms were applied in this study for the identification of similarities and differences among the investigated products.

**Figure 3:**
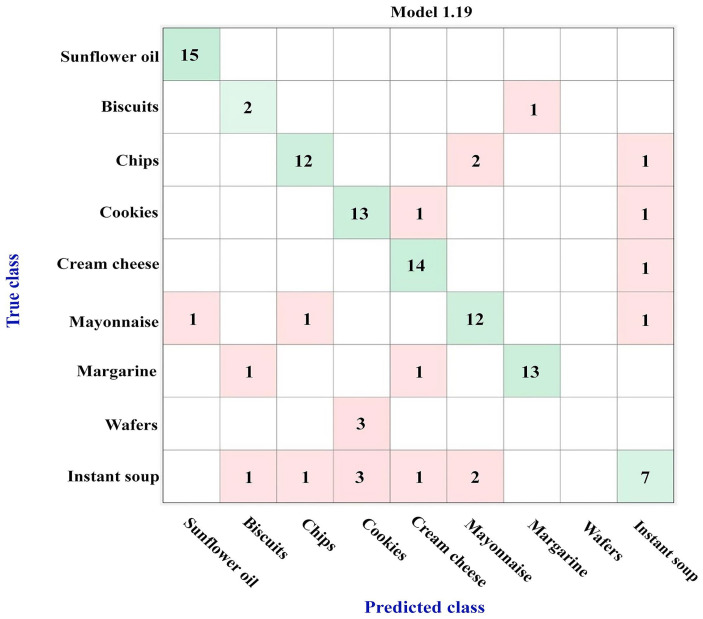
Confusion matrix of the nine types of food products rich in fatty acids, obtained using the bagged tree ensemble model (accuracy of 79.3%).

Figure 3 shows the confusion matrix obtained using the results from the GC-FID analysis and ML algorithms for the nine types of investigated food products rich in fatty acids. For this investigation, 75% of the analyzed samples were used to create the training set, while 25% of the samples were randomly selected to constitute the testing set.

These results highlight the identification of specific characteristics of the sunflower oils as well as of the cream cheese samples, as all or 14 of the 15 samples were correctly assigned to their true class. For the other classes, two or more samples were wrongly attributed to the other groups of products; as a consequence, the better model obtained, bagged tree ensemble, displayed an accuracy of only 79.3%, Figure 3.

The cookies and margarine products both possessed two wrongly assigned samples, while the mayonnaise and chips had more than three products that were not associated with their real class. Biscuits and wafers were included in the study, but due to the small number of samples, the obtained results should be viewed with caution. Most likely, the fatty acid profile of the biscuits can be considered different from the other food products, but the similarity between the wafers and cookies is real. From all the studied classes, instant soup seems to have the most similarities with five of the investigated classes; thus, 8 of the 15 samples used in the training set were misclassified, with 3 as cookies, 2 as mayonnaise, and 1 as biscuits, chips, or cream cheese.

In order to verify the performance of the model, a prediction investigation was carried out on the testing set, which contained products that were not included in the dataset employed for learning. The results, presented in Table 1, highlight good prediction for mayonnaise, cream cheese, and instant soup, while one sunflower oil sample was attributed to the mayonnaise class, a margarine product to the instant soup class, one sample of chips to margarine, and one sample of cookies to the wafer group. The most significant misclassification results were related to biscuit and wafer products; both biscuit samples were attributed to cookies, while in the case of wafers, only one of the two products was associated to the real class. However, these results can be explained by considering the low number of samples of these two classes that were involved in the study. These data are consistent with the confusion matrix shown in Figure 3.

From these results, it is interesting that even the fatty acid profiles of the instant soup class were so different in the training set, there was not any issue for the predicted samples of the testing set, while for the sunflower oils, the situation was quite different, with good assignments for the training set samples and a wrong prediction to mayonnaise for one of the five samples involved in the testing set. This similarity with the mayonnaise group for the sunflower oil products needs to be investigated in detail. Most likely, the situation is due to the fact that the sunflower oil is a raw material used for the preparation of the mayonnaise products, and thus, the wrong assignment is attributed to a derived product.

**Figure 4:**
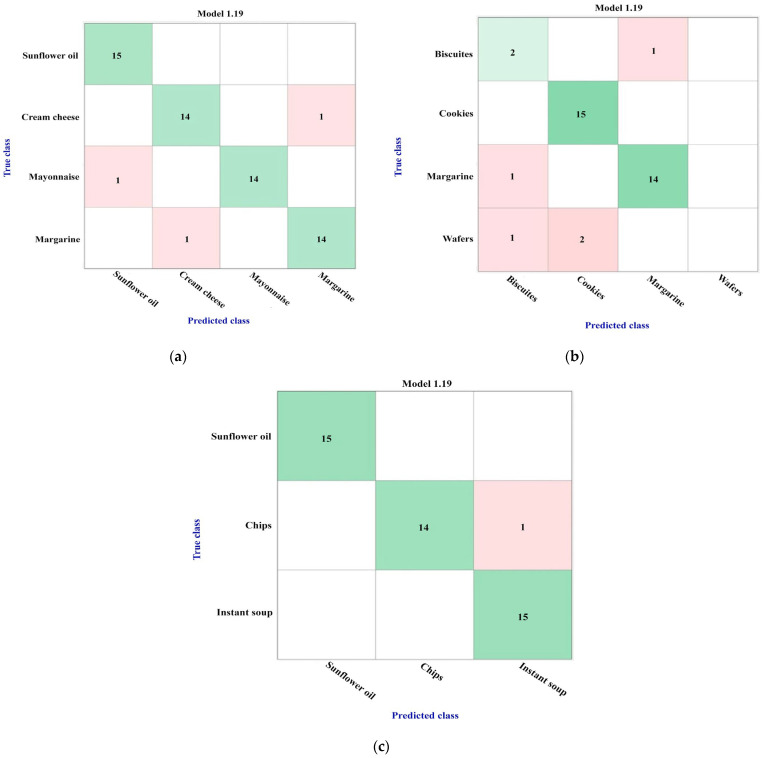
Confusion matrices obtained using the bagged tree ensemble model for: (**a**) 1st category (sunflower oil, mayonnaise margarine, and cream cheese)—accuracy of 95%; (**b**) 2nd category (biscuits, cookies, margarine, and wafers)—accuracy of 86.1%; (**c**) 3rd category (sunflower oils, chips, and instant soup)—accuracy of 97.8%.

Thus, considering the structural features of the investigated products, three groups of interest were created and evaluated: the first one contained sunflower oil, mayonnaise, margarine, and cream cheese products; the second category included samples of biscuits, cookies, margarine, and wafers; and the third one constituted sunflower oils, chips, and instant soup varieties. Figure 4 presents the results obtained, for all the three groups, when the bagged tree ensemble model was used for training; the model’s accuracies of these three investigated groups varied, as follows: 95% for the first category, 86.1% for the second one, and 97.8% for the third group.

According to these results, when the food products are grouped considering their compositional similitude, the trained models’ accuracies are significantly higher, as shown in Figure 3 and Figure 4. In each case, the model performance was further evaluated on the testing set samples, and the results are presented in Table 2, Table 3 and Table 4. This time, it is worth mentioning the correct prediction for the first group of interest (Table 2). Some misclassifications occurred in the second category, as both the biscuit and wafer samples were wrongly classified as cookies, and biscuits Table 3). The two wrong assignments as chips for the first two samples of the instant soup class might be considered controversial, but they are explained by the similarity between the chips and instant soup classes identified during the model training process, as shown in Table 4 and Figure 4c.

In order to understand which markers could be responsible for the obtained classification models, the prominent fatty acids (*w*/*w* %) were identified. Thus, based on the GC-FID results, eight different fatty acids (including three *trans* fatty acids) were found as predominant, and their distribution inside the nine types of products was evaluated by counting the number of samples that contained each fatty acid, as shown in Table 5. These results were compared with those obtained from the training and testing processes. Thus, for the first investigated group, where there are only three wrong assignments in the training process and no issues for the testing set samples (Figure 4a, Table 2), the analysis of the Table 5 results (blue-marked) highlights the significant role of the SFA and TFA compounds for the discrimination that occurred among the sunflower oil, mayonnaise, and margarine samples. It seems that palmitic, stearic, vaccenic acid, 7-*trans*-nonadecenoic acid, and 10-*trans*-nonadecenoic acid could be considered responsible for the good differentiation of these three varieties of products. For the cream cheese class, which was involved only to verify the possibilities of discrimination between fats of animal and vegetable nature, the role of *gamma*-linolenic acid must be also emphasized.

For the second category (biscuits, cookies, margarine, and wafers), where the model training accuracy was only 86.1% (Figure 4b, Table 3), the main fatty acid profile seems to be very different (orange-marked in Table 5) and confirms the supposition made above, that the low accuracy value and the wrong assignments obtained for these classes were due to the low number of samples involved of these varieties in the current study.

For the third category of fat-based products (green-marked), it appears clear that some samples of chips and instant soup classes could be assigned to one group or another since their fatty acid profile was so similar. Thus, very few differences were recorded for the palmitic, stearic, vaccenic acid, 7-*trans*-nonadecenoic acid, and 10-*trans*-nonadecenoic acid, as shown in Table 5. Most probably, these characteristics do not possess the same level of variability in all chips or instant soup samples, and, as a consequence, the issue of false assignments appeared.

**Figure 5:**
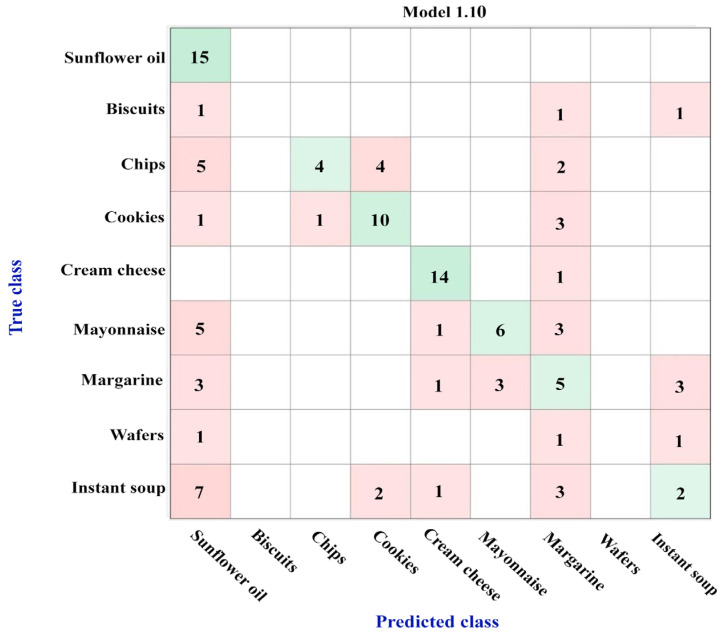
Confusion matrix for the nine types of the fat-based food products, obtained using the medium Gaussian SVM (accuracy of 50.5%) and the contents of *trans* fatty acids.

Going deeper into the analysis, we investigated the possibility of using ML algorithms and the GC-FID results for discrimination of the nine fat-based food varieties in relation to the *trans* fatty acids contained in the samples, Figure 5 and Table 6. Figure 5 shows the confusion matrix obtained in this case; it can be observed that if only *trans* fatty acid concentrations are used to classify the samples, the accuracy of the model is only 50.5%, and the possibility of successfully discriminating the products is much lower.

Low prediction results were obtained on the testing dataset too (Table 6), indicating significant similarities among the characteristic profiles of the *trans* fatty acids contained in the analyzed products; they highlight the need for using the entire fatty acid profile for good classification of the samples.

## 4. Discussion

FA composition has been investigated more or less over the years for the products we investigated.

Biscuits, which are generally offered to children, even from the youngest age, have been previously investigated, showing a fat amount varying between 2.2 and 22.8 g/100 g of the sample, with palmitic (C16:0), oleic (C18:1n-9), and linoleic (C18:2n-6) acids as the main significant FAs [27].

In some brands of potato chips found on the market in Romania, the saturated (SFA), *cis*-monounsaturated (MUFA) and polyunsaturated (PUFA) fatty acid contents have a large range of variation, namely between 6.4 and 48.8% for SFA, 41.7 and 84.5% for MUFA, and 5.5 and 28.2% for PUFA. Since potatoes possess low amounts of fat, the fat incorporation occurs during the frying process and is strongly influenced by the duration of frying [28,29].

A study reported by Brazilian researchers on various sorts of cookies (filled (28%), salty (22%), butter (12%), and wafers (4%)) indicated significant differences among the investigated brands for the total lipid amounts, the most prevalent fatty acids of the cookies being palmitic, stearic (C18:0), oleic, and linoleic acids [30]. Concerning the *trans* fatty acids (TFA), they found amounts higher than the accepted Brazilian regulation values only in one of the investigated brands of wafers. By analyzing the reported literature, they also concluded that, over the years, a gradual decrease in the *trans* fatty acid contents, both in cookies and raw materials, has occurred [30].

Cream cheese is a fresh and unripened cheese prepared from milk in the presence of lactic acid bacteria; depending on the cream cheese type, the initial fat content varies either between 4.5 and 5% or 9 and 11% [31]. Milk is a complex system containing various saturated and unsaturated fatty acids; about 98% of milk fats are triglycerides, while 2% are represented by phospholipids, cholesterol, non-esterified fatty acids, or mono and di-acyl glycerides [32]. The most significant milk fatty acids are palmitic, oleic, myristic (C14:0), and stearic acids [31].

A study realized by an Iranian research group on different mayonnaise brands commercialized in their region showed that the contents of SFAs varied between 18.1 and 24.9%, while the values of the total *cis*-unsaturated and polyunsaturated fatty acids were found to be between 68.4 and 74.4%, with palmitic, oleic and linoleic acids as the most significant saturated and unsaturated compounds of these products. For TFA, the amounts found varied from 0.6 to 3.5%, with the linoelaidic acid (C18:2 9*t*, 12*t*) as the most common of the investigated samples [33].

For the margarine category, a research group from Pakistan investigated products that exist in their market and compared them with other results from around the world [34]. They found that within this class, the SFA proportion was high (38.9–53.1%), with palmitic acid comprising the highest level of all SFA compounds (24.4–40%). The total contents of fatty acids with atherogenic potential [35], lauric (C12:0), myristic, and palmitic acids were found to be 30.9–44.3% (average of 38.4%), higher than the values reported by Danish [36] and Spanish groups [37]. For the PUFAs, they analyzed the products by dividing them into soft and hard types of margarine, indicating an average of 18.6% PUFA contents of for the soft types and a variation between 7.00 and 11.0% (mean of 8.50%) for the hard type of margarine. Regarding the TFA amounts, it seems that for the hard type, they had an average of 13.1%, which is higher than that of the soft-type products, whose average was about 3.51%. The C18:1 *trans* fatty acid was identified with higher values of 2.45–19.10%, followed by those of C18:2 *trans* fatty acids (0.80–2.00%) [34].

The composition and the content of *trans* fatty acids of various brands of chocolate wafers found in the Turkish market were previously investigated [38]. They showed that palmitic, stearic, and oleic acids were the main fatty acids determined in all chocolate wafer samples, while the *trans* fatty acid contents varied between 0.00 and 7.92%. However, the study was focused more on chocolate and chocolate wafer type.

In general, soups are recognized as good sources of various nutrients, such as minerals, vitamins, etc., and they are consumed for their health and nutritional benefits [39,40]. The variation in the free fatty acids contents during various storage periods has been realized to establish the instant soup products’ quality stability, since an increase, during storage, of the fatty acid amounts could be an indication of the hydrolytic lipolysis of the contained fat [39]. Moreover, a biological study in geriatric rats was realized for two instant soup mixtures containing lyophilized samples of chickpeas, vegetables (mushroom, parsley, dill, and celery), and some by-products (outer leaves of lettuce, onion peels, banana peels) together with olive oil and corn starch as a thickening agent [41]. The oil was added due to its nutritional and health properties, being rich in monounsaturated fatty acids and some biologically active compounds. Apart from these studies, and although instant soup is widely consumed, especially by young people, based on our knowledge, there are no other significant reported results related to the free fatty acid composition of this type of food.

Among all investigated products, sunflower oils are the most studied since they are highly used, either independently for cooking or salads or as a raw material for other fat-based products. Research performed by a Macedonian team, using the gas chromatographic method, on various types of edible oils showed mean values (% *w*/*w*) of about 8.80%, 31.50%, and 59.50% for SFAs, MUFAs, and PUFAs, respectively, with a P/S (polyunsaturated/saturated) index of 6.76 [40]. The study also highlighted a high PUFA content in sunflower seed oil (59.5%), with a prevalence of linoleic acid and a lack of linolenic acid (C18:3). The total unsaturated FA value was found to be about 91.00% in the sunflower oil, being at the highest level together with sunflower oil (92.6%).

Since the fatty acid composition is dependent on many parameters, such as species, environmental conditions, and production processes, a large variability in the determined values can be found using FA analysis for the same category of products. Moreover, considering that the FA profile affects both the physical and chemical properties of food, it is important to identify the main characteristics of each class in order to have a clear image of the product quality. Working with such large sets of data and having consistent fluctuations inside each class can represent a difficult task for food quality evaluation. Considering the complexity of the investigated matrices, as revealed by previous studies, and due to the fact that it is not possible to clearly highlight the differences among the nine classes through a simple evaluation of the fatty acid profiles or an appropriate boxplot diagram, the potential of ML algorithms for the identification of the distinct subtle changes that exist in the fatty acid profiles of the nine food products is proven by these results. Thus, the application of ML algorithms to the GC-FID data allows the development of reliable models, for an automated classification of various samples, based on their fatty acid profiles. Few studies have involved the use of ML-based methods for similar matrices. Here, it is worth mentioning the use of the fatty acid profile in combination with deep learning for edible oil adulteration [17], the identification of olive oil adulteration using fatty acid compositions and ML models [16], the prediction of fatty acid classes in snacks [42], and the authentication of oils and margarine [43].

## 5. Conclusions

The compositions of nine food categories (biscuits, chips, cookies, cream cheese, mayonnaise, margarine, wafers, instant soup, and sunflower oil) of fat-based food products were evaluated in relation to their qualitative and quantitative fatty acid contents. Due to the significant database obtained for the 150 samples involved in the study—and the difficulties of visualizing the distribution of the fatty acids—ML algorithms were employed for the analysis of the outputs.

When the samples from all nine classes were investigated using the ML methods, the better prediction model, namely the bagged tree ensemble model, showed good classification for the sunflower oil and cream cheese types, respectively. This situation appeared in the context of having sunflower oil or other appropriate products as raw materials of the other investigated classes, while cream cheese is totally different, being a matrix of animal origin. The accuracy of 79.3% for the better prediction model, obtained in this case, was the result of the lower misclassification percentages acquired for the other groups. Some of them were due to the low number of samples involved in the study, but the other results, as those recorded for instant soup, could be the consequence of the similarities found with the fatty acid profiles of biscuits, chips, cookies, cream cheese, and mayonnaise.

Considering the structural characteristics of the investigated products, three groups were created and evaluated. The bagged tree ensemble algorithm was found to possess more accurate results; thus, values of 95, 86.1, and 97.8% were observed for the first, second, and third trained groups, respectively, being lower for the category where biscuits and wafers (with a lower number of used samples) were compared. Trying to understand which markers are responsible for the obtained classification models, the prominent fatty acids were presented for each level of saturation.

## Figures and Tables

**Table 1:** Results obtained for the 9 types of fat-based products of the testing dataset by the bagged tree ensemble model (accuracy of 79.3%).

Real	Predicted	Real	Predicted
**sunflower oil**	sunflower oil	***chips***	*margarine*
**sunflower oil**	sunflower oil	**chips**	chips
***sunflower oil***	*mayonnaise*	**chips**	chips
**sunflower oil**	sunflower oil	**chips**	chips
**sunflower oil**	sunflower oil	**chips**	chips
**mayonnaise**	mayonnaise	**instant soup**	instant soup
**mayonnaise**	mayonnaise	**instant soup**	instant soup
**mayonnaise**	mayonnaise	**instant soup**	instant soup
**mayonnaise**	mayonnaise	**instant soup**	instant soup
**mayonnaise**	mayonnaise	**instant soup**	instant soup
**margarine**	margarine	***cookies***	*wafers*
**margarine**	margarine	**cookies**	cookies
**margarine**	margarine	**cookies**	cookies
***margarine***	*instant soup*	**cookies**	cookies
**margarine**	margarine	**cookies**	cookies
**cream cheese**	cream cheese	***biscuits***	*Cookies*
**cream cheese**	cream cheese	***biscuits***	*Cookies*
**cream cheese**	cream cheese	***wafers***	*Cookies*
**cream cheese**	cream cheese	**wafers**	wafers
**cream cheese**	cream cheese		

**Table 2:** Bagged tree ensemble model (95% accuracy) performance evaluated on testing set samples of the 1st category.

Real	Predicted	Real	Predicted
**sunflower oil**	sunflower oil	**margarine**	margarine
**sunflower oil**	sunflower oil	**margarine**	margarine
**sunflower oil**	sunflower oil	**margarine**	margarine
**sunflower oil**	sunflower oil	**margarine**	margarine
**sunflower oil**	sunflower oil	**margarine**	margarine
**mayonnaise**	mayonnaise	**cream cheese**	cream cheese
**mayonnaise**	mayonnaise	**cream cheese**	cream cheese
**mayonnaise**	mayonnaise	**cream cheese**	cream cheese
**mayonnaise**	mayonnaise	**cream cheese**	cream cheese
**mayonnaise**	mayonnaise	**cream cheese**	cream cheese

**Table 3:** Bagged Trees Ensemble model (86.1% accuracy) performance evaluated on testing set samples of the 2nd category.

Real	Predicted	Real	Predicted
**margarine**	margarine	**cookies**	cookies
**margarine**	margarine	**cookies**	cookies
**margarine**	margarine	**cookies**	cookies
**margarine**	margarine	***biscuits***	*cookies*
**margarine**	margarine	***biscuits***	*cookies*
**cookies**	cookies	***wafers***	*cookies*
**cookies**	cookies	***wafers***	*biscuits*

**Table 4:** Bagged Trees Ensemble model (97.8% accuracy) performance evaluated on testing set samples of the 3rd category.

Real	Predicted	Real	Predicted
**sunflower oil**	sunflower oil	**chips**	chips
**sunflower oil**	sunflower oil	**chips**	chips
**sunflower oil**	sunflower oil	***instant soup***	*chips*
**sunflower oil**	sunflower oil	***instant soup***	*chips*
**sunflower oil**	sunflower oil	**instant soup**	instant soup
**chips**	chips	**instant soup**	instant soup
**chips**	chips	**instant soup**	instant soup
**chips**	chips		

**Table 5:** The main fatty acids and the number of samples containing them that were identified inside the 9 classes of the investigated fat-based food products; for simplicity, the results were grouped according to the three investigated categories.

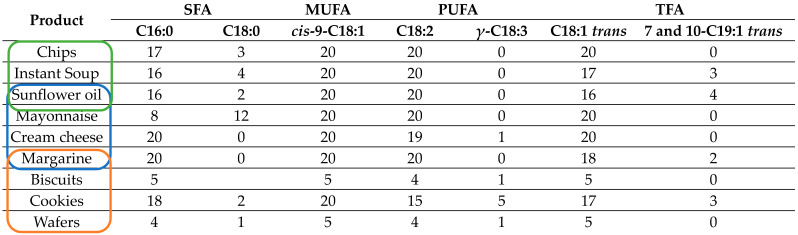

**Table 6:** Results obtained for the nine types of fat-based products of the testing dataset by considering the medium Gaussian SVM (accuracy of 50.5%) and the *trans* fatty acid GC-FID results.

Real	Predicted	Real	Predicted
**sunflower oil**	sunflower oil	***chips***	*margarine*
**sunflower oil**	sunflower oil	***chips***	*margarine*
**sunflower oil**	sunflower oil	**chips**	chips
**sunflower oil**	sunflower oil	**chips**	chips
**sunflower oil**	sunflower oil	***chips***	*margarine*
**mayonnaise**	mayonnaise	**instant soup**	instant soup
**mayonnaise**	mayonnaise	***instant soup***	*margarine*
**mayonnaise**	mayonnaise	***instant soup***	*sunflower oil*
***mayonnaise***	*sunflower oil*	***instant soup***	*sunflower oil*
**mayonnaise**	mayonnaise	***instant soup***	*sunflower oil*
***margarine***	*sunflower oil*	***cookies***	*instant soup*
**margarine**	margarine	**cookies**	cookies
***margarine***	*sunflower oil*	***cookies***	*instant soup*
***margarine***	*sunflower oil*	***cookies***	*margarine*
***margarine***	*sunflower oil*	**cookies**	cookies
**cream cheese**	cream cheese	***biscuits***	*margarine*
**cream cheese**	cream cheese	***biscuits***	*margarine*
**cream cheese**	cream cheese	***wafers***	*instant soup*
**cream cheese**	margarine	**wafers**	*margarine*
**cream cheese**	margarine		

## Data Availability

The data used to support the findings of this study can be made available by the corresponding author upon request.
